# Characterization of microsatellites and gene contents from genome shotgun sequences of mungbean (*Vigna radiata *(L.) Wilczek)

**DOI:** 10.1186/1471-2229-9-137

**Published:** 2009-11-24

**Authors:** Sithichoke Tangphatsornruang, Prakit Somta, Pichahpuk Uthaipaisanwong, Juntima Chanprasert, Duangjai Sangsrakru, Worapa Seehalak, Warunee Sommanas, Somvong Tragoonrung, Peerasak Srinives

**Affiliations:** 1 National Center for Genetic Engineering and Biotechnology, 113 Phaholyothin Rd., Klong 1, Klong Luang, Pathumthani 12120, Thailand; 2 Department of Agronomy, Faculty of Agriculture at Kamphaeng Saen, Kasetsart University, Kamphaeng Saen Campus, Nakhon Pathom 73140, Thailand

## Abstract

**Background:**

Mungbean is an important economical crop in Asia. However, genomic research has lagged behind other crop species due to the lack of polymorphic DNA markers found in this crop. The objective of this work is to develop and characterize microsatellite or simple sequence repeat (SSR) markers from genome shotgun sequencing of mungbean.

**Result:**

We have generated and characterized a total of 470,024 genome shotgun sequences covering 100.5 Mb of the mungbean (*Vigna radiata *(L.) Wilczek) genome using 454 sequencing technology. We identified 1,493 SSR motifs that could be used as potential molecular markers. Among 192 tested primer pairs in 17 mungbean accessions, 60 loci revealed polymorphism with polymorphic information content (PIC) values ranging from 0.0555 to 0.6907 with an average of 0.2594. Majority of microsatellite markers were transferable in *Vigna *species, whereas transferability rates were only 22.90% and 24.43% in *Phaseolus vulgaris *and *Glycine max*, respectively. We also used 16 SSR loci to evaluate phylogenetic relationship of 35 genotypes of the Asian *Vigna *group. The genome survey sequences were further analyzed to search for gene content. The evidence suggested 1,542 gene fragments have been sequence tagged, that fell within intersected existing gene models and shared sequence homology with other proteins in the database. Furthermore, potential microRNAs that could regulate developmental stages and environmental responses were discovered from this dataset.

**Conclusion:**

In this report, we provided evidence of generating remarkable levels of diverse microsatellite markers and gene content from high throughput genome shotgun sequencing of the mungbean genomic DNA. The markers could be used in germplasm analysis, accessing genetic diversity and linkage mapping of mungbean.

## Background

Mungbean (*Vigna radiata *(L.) Wilczek) is an important food leguminous crop in Asia, with an annual production of around 3.5 - 4.0 million tons [1]. The crop is grown principally for its protein-rich dry seeds (24% protein) which is a major protein source for people in Asian countries as part of a nutritionally balance diet [2]. It is popularly grown as a component in various cropping systems because of its ability to fix nitrogen in association with soil bacteria, early maturity (ca. 60 days) and relatively drought tolerance. Mungbean belongs to the genus *Vigna*, in which several species such as azuki bean (*Vigna angularis *(Wild.) Ohwi & Ohashi), bambara groundnut (*Vigna subterranea *(L.) Verdc.), blackgram (*Vigna mungo *(L.) Hepper), cowpea (*Vigna unguiculata *(L.) Walp.), moth bean (*V. aconitifolia *(Jacq.) Maréchal) and rice bean (*Vigna umbellata *(Thunb.) Ohwi & Ohashi), are domesticated and utilized in a similar way to mungbean.

Mungbean is a self-pollinated diploid plant with 2n = 2x = 22 chromosomes and a genome size of 515 Mb/1C [3]. Genomic study in this crop is far behind other legume crops. Mungbean was among the primary crops that genetic linkage maps have been developed. However, the current linkage maps, based on RFLP and RAPD markers of mungbean, do not resolve 11 linkage groups [4]. Microsatellites or simple sequence repeats (SSRs) are markers of choice for crop improvement of many species because they are reliable and easy to score [5]. SSRs are clusters of short tandem repeated nucleotide bases distributed throughout the genome. SSR markers are co-dominant, multi-allelic and requiring small amount of DNA for scoring. The traditional method of SSR marker development involves construction of SSR-enriched library, cloning, and sequencing, which is costly and labor intensive. Nevertheless, significant efforts have been invested in development of SSR markers in recent years, but so far only 35 polymorphic SSR markers published for mungbean [6-10]. In a study by Somta *et. al*. (2008), more than 200 primer pairs amplifying SSRs were tested for polymorphism among 17 mungbean accessions, only 12 (5.7%) primer pairs were polymorphic. The authors suggested that the use of SSR markers has been limited due to the lack of polymorphism in this species [7].

Over the past few years, the introduction of a massively-parallel pyrosequencing technology developed by 454 Life Sciences Technology has opened new possibilities for high-throughput genome analysis [11]. This new technology has been applied to the sequencing of microbial genomes, genotyping, genome resequencing, transcriptome profiling and methylation studies. Although, sequences generated by this technique are relatively short, there are evidences suggesting that this technique can be used to sequence plant genomes that are complex and large [12-14]. Wicker *et al*. (2006) suggested that 454 sequencing technology could reveal almost complete assembly of the entire gene sequences in 4 barley BAC clones at only 9-folds coverage and concluded that the method is a rapid and cost effective way of sequencing the gene-containing portions of the genome. Low coverage shotgun sequencing using 454 sequencing technology has also been used to study functional genomics in soybean [12], repetitive DNA in the pea genome [14] and transcriptome from a normalized cDNA library of *Medicago truncatula *[15]. Here, we report genome shotgun sequencing of the mungbean genomic DNA using 454 Life Sciences sequencing technology for isolation of SSR markers and characterization of gene content.

## Results and Discussion

### Shotgun sequencing of *Vigna radiata *genome

Sequencing of *Vigna radiata *genomic DNA was carried out using 454 Life Sciences technology on the Genome Sequencer (GS) FLX System. A total of 470,024 quality filtered sequence reads was generated with the average read length of 216 bases covering 100.5 Mb. All reads were deposited in NCBI Short Read Archive (ID = SRA003681) http://www.ncbi.nih.gov/Traces/. Assembly of the obtained nucleotide sequence reads was performed using the Newbler, de novo sequence assembly software [11]. Redundant reads were reduced to 46,646 contigs with the average contig length of 297 bases covering 13.85 Mb. The contig sequence data were reported in the DDBJ/EMBL/GenBank nucleotide sequence databases with the accession number BABL01000001-BABL01046645. The contig length ranges from 89 bases to 44,462 bases. The average GC content of mungbean genomic DNA generated in this study is 34.69% which is consistent with the reports on GC contents in other plant genomes such as *Arapbidopsis *(36% [16]), grape (34.6% [17]), poplar (33.7% [18]), tomato (36.2% [19]) and potato (35.6% [19]). It is slightly higher than the mean of GC content for intergenic regions in the *Arabidopsis *genome (32.9%, Genome Indices 8/04: http://http//gi.kuicr.kyoto-u.ac.jp) [20]; but it is much lower than the average GC content of *Arabidopsis *coding sequences (44.5%) [21].

### Characterization of polymorphic microsatellite markers in *Vigna radiata*

We isolated 1,493 microsatellite regions using the Troll software. There were 889 dinucleotide repeats (DNPs), 282 trinucleotide repeats (TNPs), 123 tetranucleotide repeats (TTNPs), 124 pentanucleotide repeats (PNPs) and 75 SSRs with hexanucleotide repeats or more. The distribution of the number of motif repeat ranged from 4 - 30 repeats (Table 1). The most common motif type of DNPs was TA/AT (89.3% of DNPs) followed by TC/AG (7.1% of DNPs) and AC/TG (3.6% of DNPs). The GC/CG motif was not found in the data set. TNPs were found at 282 SSR loci (18.9%), which was three times lower than that of DNPs. The TAA repeat was the most common motif type found at 184 loci (65.24% of TNPs). The least frequent TNP motif was GC-rich (GCG/CGC) found at only 2 loci. The genomic SSRs with GC-rich motif repeats are rare in most plants as previously reported in rice, corn, soybean [22], wheat [23], *Arabidopsis thaliana*, apricot, peach [24], coffee [25] and rubber tree [26]. In contrast, the GC-rich motifs have been reported as frequent motifs in studies on development of SSR from expressed sequence tags and genomes with methylation filtration [27-30]. Thus, GC-rich SSR are most likely to be derived from the coding region of the genome. The frequency of identified SSR in mungbean was one SSR in every 67 kb (1,493 SSRs in 100.5 Mb) which is significantly lower than the SSR frequency in soybean (1/7.4 kb) [31]. Among plant species, the SSR frequencies range from 1/1.5 kb in coffee to 1/20 kb in cotton [25,31]. The observed low SSR frequency in this study is probably because a large proportion of reads from the low coverage sequencing (0.2x) of the mungbean genome were biased toward highly repetitive parts of the genome.

**Table 1:** Distribution of identified SSRs using the Troll software according to SSR motif type and repeat number.

Number of motif repeat	Di	Tri	Tetra	Penta	Hexa	Hepta	Octa
**n = 4**	N/A	N/A	N/A	97	45	14	2
**n = 5**	N/A	N/A	89	20	4	2	0
**n = 6**	N/A	N/A	23	4	3	1	0
**n = 7**	N/A	142	9	2	0	0	0
**n = 8**	N/A	75	1	0	0	0	0
**n = 9**	N/A	36	0	0	0	1	1
**n = 10**	137	17	1	1	1	0	1
**n = 11**	115	4	0	0	0	0	0
**n = 12**	80	3	0	0	0	0	0
**n = 13**	50	1	0	0	0	0	0
**n = 14**	59	0	0	0	0	0	0
**n = 15**	50	2	0	0	0	0	0
**n = 16**	46	1	0	0	0	0	0
**n = 17**	45	0	0	0	0	0	0
**n = 18**	49	1	0	0	0	0	0
**n = 19**	43	0	0	0	0	0	0
**n = 20**	48	0	0	0	0	0	0
**n = 21**	32	0	0	0	0	0	0
**n = 22**	25	0	0	0	0	0	0
**n = 23**	30	0	0	0	0	0	0
**n = 24**	34	0	0	0	0	0	0
**n = 25**	10	0	0	0	0	0	0
**n = 26**	19	0	0	0	0	0	0
**n = 27**	10	0	0	0	0	0	0
**n = 28**	3	0	0	0	0	0	0
**n = 29**	3	0	0	0	0	0	0
**n = 30**	1	0	0	0	0	0	0

**total**	**889**	**282**	**123**	**124**	**53**	**18**	**4**

From 1,493 identified SSRs, 192 SSRs were identified from contigs and 1,301 SSRs were from singletons. Among 192 contigs containing SSR motifs, majority of contigs were assembled from 2 reads (87 contigs) followed by 3 reads (48 contigs) and 4 reads (16 contigs) (Table 2). By applying the Lander-Waterman model [32] to this dataset, there should be no contig assembled from more than 9 reads provided that all sequences were generated by chance from non repetitive DNA (Table 2). Therefore, 16 out of 192 contigs that were assembled from more than 9 reads are likely to represent repetitive sequences of the genome. It should be noted that loci present in multiple copies are not desirable for construction of genetic maps. Interestingly, there was a highly repetitive contig containing SSR (contig 44495) which was assembled from 3,174 raw reads. Sequence homology search revealed that contig 44495 is a fragment of the chloroplast genome. The number of chloroplast genome of higher plants can reach hundreds of copies per cell. Due to the deep sequencing nature of 454 technology, it is expected to obtain a large number of reads from sequences with multiple copies such as organellar genomes, transposons and ribosomal DNA [12]. The degree of sequencing over-representation in a repetitive genome can be estimated from the difference between the observed read coverage and the predictions from the Lander-Waterman model (Table 2) as suggested by [12]. It should be noted that the number of observed contigs with assembled reads = 2 was much lower than the prediction by the model. This was probably due to the effect of low sequencing coverage; thus it was not included in the calculation of the number of repetitive reads. In total, there were 241,410 reads (51%) present in multiple copies. We estimated that 51% of shotgun reads from 0.2× genome coverage represented repetitive DNA. This estimate is slightly more than the result from the DNA re-association kinetic study which estimated 46% of the total leaf DNA as repetitive sequences [33].

**Table 2:** The table lists number of contigs containing SSRs, observed number of contigs from 454 data set, predicted number of contigs according to the Lander-Waterman model for sampling a completely non-repetitive genome and the repetitive sequences calculated using the differences between the observed number of contigs and the predictions.

Number of reads in contigs	Predicted number of contigs by model	Observed number of contigs	repetitive reads (observed-predicted)	Observed number of contigs containing SSR
2	59398	19622	n/a	87
3	10350	13576	9678	48
4	1803	6020	16868	16
5	314	2491	10885	6
6	55	1270	7290	10
7	10	765	5285	2
8	2	518	4128	3
9	1	363	3258	4
10	0	262	2620	1
11	0	216	2376	2
12	0	169	2028	1
13	0	149	1937	1
14	0	116	1624	1
15	0	115	1725	3
≥ 16	0	994	171708	7

total			241410	192

To evaluate these SSR loci in further detail, we designed 192 primer pairs to amplify all SSR loci identified from the contig data set. Among the 192 primer pairs evaluated in 17 mungbean accessions, 179 (93.23%) primer pairs were amplifiable and 127 (66.14%) primer pairs produced scorable bands. Of these, 58 primer pairs targeting 60 loci revealed polymorphism because 2 primer pairs, VR257 and VR400, were able to target 2 independent loci for each primer pair. Characteristics of all 60 loci are summarized in Additional File 1. These primer pairs were able to detect a range of 2 to 6 alleles with a mean of 2.6833 alleles per locus. Polymorphic information content (PIC) values ranged from 0.0555 to 0.6907 with an average of 0.2594 which is similar to the previous studies [7,34]. In this study, there were 33 pair-wise combinations that significantly deviated from linkage disequilibrium (LD). Genetic variation at a given locus in a population is measured by the observed heterozygosity (*H*_O_). The *H*_O _values varied from 0 to 0.6471 with the average *H*_O _of 0.0289; while the expected heterozygosity (*H*_E_) values ranged from 0.0571 to 0.7356 with the average *H*_E _of 0.2908. Tests for Hardy-Weinberg equilibrium (HWE) of the polymorphic loci revealed that all loci, except VR400, were significantly deviated from HWE (*P *< 0.05). This is in agreement with the previous studies in mungbean which have shown that most if not all of the loci deviated from HWE [6,7,34]. The low level of heterozygosity and significant deviation from HWE are probably because mungbean is a highly self-pollinated species with an estimated outcrossing rate of only 1.1% [35].

We also tested the SSR locus in the highly repetitive contig 44495, which was a fragment on the chloroplast genome. The VR0453 locus, located in the non-coding region near the *atpB *gene in the chloroplast genome, had 2 alleles and showed relatively low PIC value of 0.1046 (see Additional File 1). Chloroplast microsatellites have been used in ecological and evolutionary studies, especially at the intraspecific level, because they are nonrecombinant, uniparentally inherited and effectively haploid [36]. However, the major barrier for utilization of chloroplast microsatellites is the low mutation rates associated with the chloroplast genome [37] leading to low polymorphism level of markers in the chloroplast genome.

Sequence homology search of other loci against the Genbank non-redundant protein database and the TIGR plant repeat databases [38] revealed that there were 5 loci (VR029, VR073, VR216, VR256 and VR323) matched unknown proteins, 1 locus (VR390) matched beta-glucosidase and 1 locus (VR102) matched pectinesterase (see Additional File 1). Note that there was no sequence matched against known repeat sequences in the TIGR plant repeat databases.

### Cross-species transferability of *Vigna radiata *microsatellite markers

**Figure 1:**
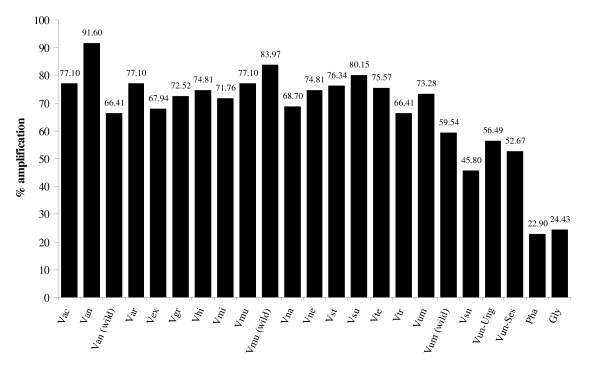
**Cross-species amplification of 127 mungbean microsatellite markers in various species from genus *Vigna*, *Phaseolus *and *Glycine***. Abbreviations are as followed: Vac = *V. aconitifolia*, Van = *V. angularis *var. *angularis*, Van (wild) = *V. angularis *var. *nipponensis*, Var = *V. aridicola*, Vex = *V. exilis*, Vgr = *V. grandiflora*, Vhi = *V. hirtella*, Vmi = *V. minima*, Vmu = *V. mungo *var. *mungo*, Vum(wild) = *V. mungo *var. *sylvestris*, Vna = *V. nakashimae*, Vne = *V. nepalensis*, Vra = *V. radiata *var. *radiate*, Vra(wild) = *V. radiata *var. *sublobata*, Vst = *V. stipulacea*, Vsu = *V. subramaniana*, Vte = *V. tenuicaulis*, Vtr = *V. trilobata*, Vum = *V. umbellate*, Vsn = *V. subterranean*, Vun-Ung = *V. unguiculata *cv-gr. *Unguiculata*, Vun-Ses = *V. unguiculata *cv-gr. *Sesquipedalis*, Pha = *P. vulgaris *and Gly = *G. max*.

With the exception of azuki bean (*V. angularis*), SSR markers are very limited for other *Vigna *species. Therefore, novel markers with high cross-species transferability rates are desirable. Cross-species amplification of the 127 microsatellite markers was assessed in 24 taxa of legumes in the tribe *Phaseoleae *including genus *Vigna *(African and Asian *Vigna*), *Phaseolus *and *Glycine*. One hundred and twenty five primer pairs successfully amplified DNA from more than one legume. Five primer pairs were able to amplify DNA of all legume taxa tested; while VR339 amplified only 1 legume species, *V. aconitifolia*. In most cases, mungbean microsatellite primers were able to amplify DNA of other *Vigna *species (Figure 1). The transferability rates of mungbean primers were between 45.80% (*V. subterranean*) and 91.60% (*V. angularis*). However, the amplification rate was reduced in *Phaseolus vulgaris *and *Glycine max *to 22.90% and 24.43%, respectively (Figure 1). Transferability rate of mungbean genomic microsatellite markers to other *Vigna *species appeared to be more or less similar to previous studies. Somta et al. (2009) reported that amplification of genic microsatellite markers in 19 taxa of *Vigna *species was between 80% (*V. aconitifolia*) to 95.3% (*V. reflex-pilosa*) [39]. Whereas, Chaitieng et al. (2006) reported that amplification of azuki bean (*V. angularis*) microsatellite markers in *V. mungo*, *V. radiata*, *V. aconitifolia *and *V. umbellata *was between 68.8 to 90.2% [40]. The high amplification rates of both mungbean and azuki bean microsatellite markers in *Vigna *species indicate high genome homology among species in this genus and are useful for genetics and genomics studies, especially genome mapping and comparative genomics.

### Phylogenetic relationship

**Figure 2:**
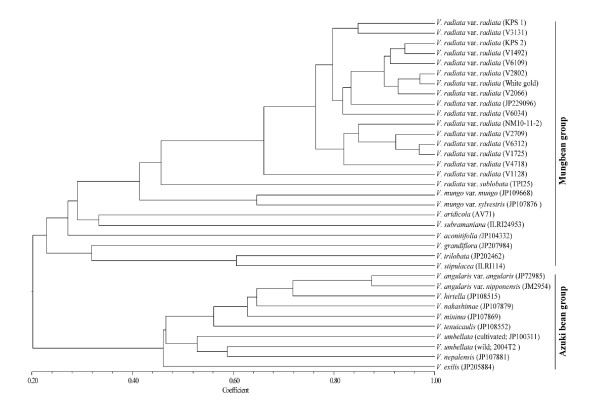
**A dendrogram depicting genetic diversity and relationships among 35 genotypes from 20 taxa of Asian *Vigna *as revealed by the polymorphism of 16 mungbean microsatellite markers**. Accession codes from the AVRDC-The world vegetable center and the National Institute of Agrobiological Sciences (Japan) are provided in brackets.

To determine the genetic diversity structure and relationships between 35 genotypes of 20 taxa of Asian *Vigna*, polymorphism scores at 16 microsatellite loci without missing data were used (see Additional File 2). UPGMA cluster analysis was conducted using software NTSYSpc 2.2 [41]. Results from the cluster analysis revealed that all the genotypes of Asian *Vigna *could be clearly differentiated and classified into two groups; mungbean group and azuki bean group (Figure 2). The results were in agreement with previous studies using non-coding sequences of *trn*T-F [42,43]. In contrast, studies using AFLP [44], rDNA-ITS and *atp*B-*rbc*L sequences [45] recognized three groups within the Asian *Vigna*. In addition, it is worth noting that *V. nepalensis*, which has similar morphology [46] and close genetic relationship with *V. angularis *[43,45], was found to be highly distinct in our study. *V. grandiflora *previously shown to have high morphological and genetic similarity to *V. radiata *[46,47] was found to have closer genetic relationship with *V. trilobata *and *V. stipulacea *than *V. radiate *in this study. Also, *V. subramaniana *that was reported to be closely related to mungbean [45] appeared to be more distant from mungbean but more closely related to *V. aridicola *in our study. It should be noted that *V. subramaniana *has a complex taxonomic history, controversy in the literature and classification concerning the taxonomy of this species still remains [48]. The differences in the phylogenetic relationship of Asian *Vigna *may be explained by the differences in the methods used in the previous studies. Morphological traits [46], rDNA and cpDNA sequences [43,45] were used in previous studies to demonstrate phylogenetic relationship, while our study used SSR markers for demonstration. The use of PCR-based SSR markers may possibly result in size homoplasy of PCR products between/among species [49]. The same allele size of an SSR locus may contain different sequence variants; thus species sharing the same SSR allelic size include species that are identical by descent and species that have originated from convergent evolution.

### Sequence annotation and gene ontology

**Figure 3:**
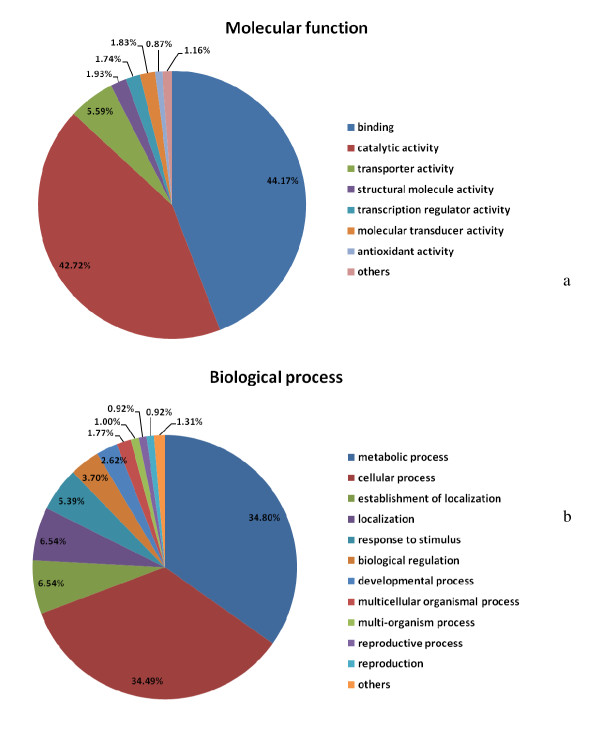
**Gene Ontology classification of the predicted mungbean ORFs according to molecular function (a) and biological process (b) using BLAST2GO **[51]**with E-6 cutoff**.

The contigs were analyzed by GeneMark.hmm eukaryotic version 3.3 [50] to predict Open Reading Frame (ORF) using *Medicago trunculata *as a model organism and default parameter conditions. Results from GeneMark predicted a total of 44,112 ORFs. For functional annotation, the potential coding regions were analyzed by BLAST2GO [51] leading to consistent gene annotations, assigning gene names, gene products, EC numbers and Gene Ontology (GO) numbers. Gene Ontology provides a system to categorize description of gene products according to three ontologies: molecular function, biological process and cellular component. Sequence homology search revealed that there were 1,542 ORFs matches with non-redundant protein database with an E-value cut-off at E-6. Nine hundreds and fifty sequences were mapped to one or more ontologies with multiple assignments possible for a given protein within a single ontology. There were 647 assignments made to the molecular function ontology, with a large proportion of these in catalytic (42.72%) and binding activities (44.17%) categories (Figure 3a). Under the biological process ontology, 555 assignments were made with a large proportion of assignments fell into metabolic process and cellular process (such as secretory pathway, transcription and translation) categories (Figure 3b).

### Similarity of mungbean predicted ORFs with other plant ESTs

**Figure 4:**
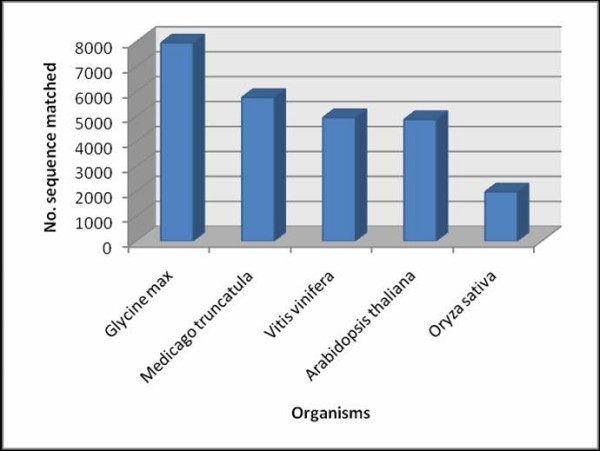
**Comparison of mungbean ORFs with 8 other plant gene indices by tBLASTX (e-value cutoff = E-6)**. Blue bars represent mungbean contigs with similar homology search against other plant gene index databases including soybean (GMGI, 13.0), Arabidopsis (AGI, 13.0), rice (OGI, 17.0), *M. truncatula *(MTGI, 9.0) and *Vitis vinifera *(VVGI, 6.0).

To identify gene functions, the mungbean contig set was blasted (TBLASTX) to identify ESTs encoding similar proteins, at an e-value cutoff at E-6, against other plant gene indices collected in The Gene Index Databases, Dana Farber Cancer Institute, such as soybean (GMGI, 13.0), Arabidopsis (AGI, 13.0), rice (OGI, 17.0), *M. truncatula *(MTGI, 9.0) and *Vitis vinifera *(VVGI, 6.0) [52]. The number of sequences that showed similarity to encoding sequences is shown in Figure 4. Comparison between the mungbean dataset and the *Glycine max *gene index gave the highest number of matched sequences (7,940 sequences). *V. radiata *and *G. max *are grouped together as tropical season legumes or Phaseoloid exhibiting extensive genome conservation based on previous comparative genetic mapping [53,54]. The other Papilionoideae legume, *M. truncatula*, which is a cold season legume, also shares a large number of homologous sequences (5,759 sequences) with the mungbean dataset. *A. thaliana *and *V. vinifera *gave lower number of matched sequences to the mungbean dataset; 4872 and 4,949 sequences respectively. The lowest number of matched sequences (1,971 sequences) was observed when the mungbean dataset was blasted against the *Oryza sativa *gene index, the only monocot plant used in the comparison.

### Discovery of microRNA

To predict functional non-coding RNA, such as microRNA, in the mungbean dataset, we made computational prediction of potential microRNA using MiRFinder to search for the potential hairpin-loop structure in their sequences [55]. Next we calculated the minimal folding free energy (MFE) using Sfold [56]. There were 2,247 microRNA candidates with MFE < -25 kcal/mol which were selected for further analysis. Then we blasted the mungbean microRNA candidates against previously known microRNAs from Arabidopsis, rice, and other plant species to search for potentially conserved microRNAs. A total of 4 miRNA candidates had sequence homology with miR171, miR408, miR1171 and miR414, which have been shown to target genes coding for SCARECROW-like proteins implicated in radial root pattern [57], plantacyanin [58], copper chaperone [59] and translation initiation factor [60], respectively (Table 3).

**Table 3:** Results from homology search of the mungbean microRNA candidates against the microRNA database.

Read name	Contig	miRNA family	MFE (kcal/mol)	Target	Ref
E4UUDJH02I4UG8	contig25352	miR171	-37	SCARECROW-like protein	Reinhart et al., 2002
E4UUDJH02HINHM	contig16040	miR408	-29	plantacyanin	Sunkar and Zhu, 2004
E4UUDJH01ECGTX	contig11544	miR1171	-25	putative copper chaperone3	Molnar et al., 2007
E4UUDJH01AVSEX	contig25342	miR414	-26	TIF3H1	Fattash et al., 2007

microRNA families and their target genes are also present in the table.

## Conclusion

The results provided by the present study highlight a reliable and efficient way in obtaining polymorphic microsatellite markers and characterization of putative genes using shotgun genome sequences of *Vigna radiata*. A significance of the results from this study is that high-throughput shotgun sequences of mungbean can be useful not only for marker development, construction of linkage map, mungbean genetic improvement, phylogenetic relationship, but also for gene discovery as the paucity of DNA markers in cultivated mungbean has precluded detailed genetic research on this crop.

## Methods

### Plant materials, DNA extraction and 454 Life Sciences Sequencing

Seventeen accessions of mungbean (*Vigna radiata*) and the other 23 taxa of legumes in the tribe *Phaseoleae *including genus *Vigna *(African *Vigna *and Asian *Vigna*), *Phaseolus *and *Glycine *as listed in Additional File 3 were used in this study. For sequencing, DNA was extracted from young leaf tissue of mungbean cultivar "Kamphaeng Saen 1" using DNAeasy Plant Mini Kit (Qiagen). For SSR analysis, DNA of all plant materials was extracted from young fresh leaves using CTAB method [61]. The concentration of each sample was calculated from OD measurement and the samples were separated by gel electrophoresis on 0.8% agarose gels. The sequencing was performed using the GS-FLX instrument (454 Life Sciences, Branford, CT) and yielded 470,024 quality filtered sequence reads with the average length of 216 bp. The reads were deposited into NCBI Short Read Archive.

Prediction of sequencing coverage in contigs from a completely non repetitive genome was calculated according to the Lander and Waterman model [32]. The number of contigs expected containing a number of reads j is given by equation 1.

Where *N *is the number of reads, *L *is the read length, *G *is the haploid genome size in base pairs, and *T *is the base pair overlap required for contig formation (in this case *T *= 40).

### Isolation, amplification and transferability of SSR markers

In order to identify microsatellite markers, non-redundant sequences were screened for SSRs using TROLL software http://wsmartins.net/webtroll/troll.html. For the searches, we defined SSRs as being DNP ≥ 14 bases; TNP ≥ 15 bases; TTNP ≥ 16 bases; HNP (and more) ≥ 16 bases [31]. For comparison of SSRs in plant genomic sequences, we used the criteria of SSR motif of ≥ 20 bases [31,62,63]. Primer pairs were designed to amplify microsatellite regions using PRIMER3 [64]. PCR was carried out in a total volume of 10 μL containing 2 ng of DNA template, 1× *Taq *buffer, 2 mM MgCl_2_, 0.2 mM dNTPs, 1 U *Taq *DNA polymerase (Fermentas) and 0.5 μM each of forward and reverse primers. Amplification was performed in a GeneAmp PCR 9700 System thermocycler (Applied Biosystems) programmed as follow: 94°C for 2 min followed by 35 cycles of 94°C for 30 s, 50-65°C for 30 s, 72°C for 1 min, and a final extension step at 72°C for 10 min. Amplified products were separated on 5% denaturing polyacrylamide gels and visualized by silver-staining.

### Analysis of polymorphic loci

Seventeen mungbean genotypes as listed in Additional File 3 were used for polymorphism analysis of SSR markers. Details of primer pairs for SSR markers are listed in Additional File 4. Scoring data from polymorphic loci were used to calculate Polymorphism Information Content (PIC) [65], Hardy-Weinberg equilibrium (HWE) [66], pairwise linkage disequilibrium (LD) using chi-square test, and observed heterozygosity and expected heterozygosity using the PowerMarker 3.25 software [67].

### Cross taxa transferability and phylogenetic relationship

The cross taxa transferability of all scorable 127 SSR loci was evaluated using 17 accessions of mungbean (*Vigna radiata*) and the other 23 taxa of legumes in the tribe *Phaseoleae *including genus *Vigna *(African *Vigna *and Asian *Vigna*), *Phaseolus *and *Glycine *(see Additional File 3). The percentage of transferability was calculated for each taxon (23 taxa) in which the detected fragment/the total number of loci analyzed. A genetic similarity matrix (see Additional File 2) was prepared for 35 genotypes from 20 taxa at 16 SSR loci (as listed in Additiional File 4). UPGMA (unweighted pair group method with arithmetic mean) cluster analysis was conducted using software NTSYSpc 2.2 [41].

### Analysis of gene content and annotation

The mungbean contig set was analyzed in two parts which are 1) gene prediction/Gene Ontology (GO) term annotation and 2) functional gene identification. GeneMark.hmm eukaryotic version 3.3 [50] based on Hidden Markov Models was used to predict coding sequence (cds) of the contig set using *Medicago trunculata *as a model organism and default parameter conditions. For the functional annotation, the potential coding sequences were analyzed by BLAST2GO [51]. To identify gene functions, sequence similarity search program-BLAST was used to identify ESTs encoding similar proteins of the mungbean contig set. All 46,646 contigs were blasted (TBLASTX) with the threshold E-value cutoff at 1e-6 against 580,213 assembled Unique Transcripts sequences from various plant species from The Plant Genome DataBase (PlantGDB) [52], which included *Arabidopsis thaliana *(324,630), *Glycine max *(105,862), *Medicago truncatula *(57,231), *Oryza sativa *(44,644), and *Vitis vinifera *(47,846).

## Authors' contributions

ST conceived of the study together with the other authors, carried out the major part of the experiments, analyzed the results and drafted the manuscript. PS, WS and WM prepared plant materials and performed genetic analysis. DS participated in library construction and sequencing. PU and JC participated in analysis of the results. PS and ST participated in coordination and analysis of the results. All authors participated in writing the final manuscript. All authors read and approved the final manuscript.

## Supplementary Material

Additional file 1 **Characteristics of 58 primer pairs targeting 60 polymorphic microsatellite loci analyzed in 17 accessions of mungbean (16 cultivated and 1 wild mungbean) as listed in Additional File **3. PCR conditions and electrophoresis were described in Somta et al. (2008) [7]. Polymorphism information content (PIC), observed heterozygosity, expected heterozygosity, Hardy-Weinberg equilibrium (HWE), pair-wise and linkage disequilibrium (LD) of polymorphic loci were calculated using software POWERMARKER 3.25 [67]. Click here for file

Additional file 2 **Dice's Similarity Matrix**. This additional file contains a table expressing the Dice's Similarity Matrix. Click here for file

Additional file 3 **Plants used in this study**. This additional file contains a table listing all of the plants used in the study. Click here for file

Additional file 4 **SSRs used in the phylogenetic study**. This additional file contains a table showing the SSRs used in the phylogenetic study. Click here for file

## References

[B1] Weinberger K Impact analysis on mungbean research in south and southeast Asia AVRDC Processing No 9991175. Shanhua, Taiwan 2003

[B2] Poehlman JM The mungbean 1991 New Delhi: Oxford & IBH Publishing Co. PVT. Ltd

[B3] Parida A Raina SN Narayan RKJ Quantitative DNA variation between and within chromosome complements of *Vigna *species (Fabaceae) Genetica 1990 82 125 133 10.1007/BF00124642

[B4] Humphry E Konduri V Lambrides J Magner T McIntyre L Aitken B Liu J Development of a mungbean (*Vigna radiata*) RFLP linkage map and its comparison with lablab (*Lablab purpureus*) reveals a high level of colinearity between the two genomes Theor Appl Genet 2002 105 1 160 166 10.1007/s00122-002-0909-1 12582573

[B5] Gupta P Varshney R The development and use of microsatellite markers for genetic analysis and plant breeding with emphasis on bread wheat Euphytica 2000 113 163 185 10.1023/A:1003910819967

[B6] Gwag JG Chung JW Chung HK Lee JH Ma KH Dixit A Park YJ Cho EG Kim TS Lee SH Characterization of new microsatellite markers in mung bean, *Vigna radiata *(L.) Molecular Ecology Notes 2006 6 4 1132 1134 10.1111/j.1471-8286.2006.01461.x

[B7] Somta P Musch W Kongsamai B Chanprame S Nakasathien S Toojinda T Sorajjapinun W Seehalak W Tragoonrung S Srinives P New microsatellite markers isolated from mungbean (*Vigna radiata *(L.) Wilczek) Mol Ecol Resource 2008 8 1155 1157 10.1111/j.1755-0998.2008.02219.x 21586000

[B8] Kumar SV Tan SG Quah SC Yusoff K Isolation of microsatellite markers in mungbean, *Vigna radiata *Molecular Ecology Notes 2002 2 2 96 98 10.1046/j.1471-8286.2002.00158.x

[B9] Kumar SV Tan SG Quah SC Yusoff K Isolation and characterization of seven tetranucleotide microsatellite loci in mungbean, *Vigna radiata *Molecular Ecology Notes 2002 2 3 293 295 10.1046/j.1471-8286.2002.00239.x

[B10] Miyagi M Humphry M Ma ZY Lambrides CJ Bateson M Liu CJ Construction of bacterial artificial chromosome libraries and their application in developing PCR-based markers closely linked to a major locus conditioning bruchid resistance in mungbean (*Vigna radiata *L. Wilczek) Theor Appl Genet 2004 110 1 151 156 10.1007/s00122-004-1821-7 15490104

[B11] Margulies M Egholm M Altman WE Attiya S Bader JS Bemben LA Berka J Braverman MS Chen YJ Chen Z Genome sequencing in microfabricated high-density picolitre reactors Nature 2005 437 7057 376 380 16056220 10.1038/nature03959 PMC1464427

[B12] Swaminathan K Varala K Hudson ME Global repeat discovery and estimation of genomic copy number in a large, complex genome using a high-throughput 454 sequence survey BMC Genomics 2007 8 132 10.1186/1471-2164-8-132 17524145 PMC1894642

[B13] Wicker T Schlagenhauf E Graner A Close TJ Keller B Stein N 454 sequencing put to the test using the complex genome of barley BMC Genomics 2006 7 275 10.1186/1471-2164-7-275 17067373 PMC1633745

[B14] Macas J Neumann P Navratilova A Repetitive DNA in the pea (*Pisum sativum *L.) genome: comprehensive characterization using 454 sequencing and comparison to soybean and Medicago truncatula BMC Genomics 2007 8 427 10.1186/1471-2164-8-427 18031571 PMC2206039

[B15] Cheung F Haas BJ Goldberg SM May GD Xiao Y Town CD Sequencing Medicago truncatula expressed sequenced tags using 454 Life Sciences technology BMC Genomics 2006 7 272 10.1186/1471-2164-7-272 17062153 PMC1635983

[B16] Analysis of the genome sequence of the flowering plant *Arabidopsis thaliana *Nature 2000 408 6814 796 815 10.1038/35048692 11130711

[B17] Jaillon O Aury JM Noel B Policriti A Clepet C Casagrande A Choisne N Aubourg S Vitulo N Jubin C The grapevine genome sequence suggests ancestral hexaploidization in major angiosperm phyla Nature 2007 449 7161 463 467 10.1038/nature06148 17721507

[B18] Tuskan GA Difazio S Jansson S Bohlmann J Grigoriev I Hellsten U Putnam N Ralph S Rombauts S Salamov A The genome of black cottonwood, *Populus trichocarpa *(Torr. & Gray) Science 2006 313 5793 1596 1604 10.1126/science.1128691 16973872

[B19] Zhu W Ouyang S Iovene M O'Brien K Vuong H Jiang J Buell CR Analysis of 90 Mb of the potato genome reveals conservation of gene structures and order with tomato but divergence in repetitive sequence composition BMC Genomics 2008 9 286 10.1186/1471-2164-9-286 18554403 PMC2442093

[B20] Thomas BC Rapaka L Lyons E Pedersen B Freeling M Arabidopsis intragenomic conserved noncoding sequence Proc Natl Acad Sci USA 2007 104 9 3348 3353 10.1073/pnas.0611574104 17301222 PMC1805546

[B21] Wang HC Hickey DA Rapid divergence of codon usage patterns within the rice genome BMC Evol Biol 2007 7 Suppl 1 S6 10.1186/1471-2148-7-S1-S6 17288579 PMC1796615

[B22] Gao LF Tang JF Li HW Jia JZ Analysis of microsatellites in major crops assessed by computational and experimental approaches Molecular Breeding 2003 12 3 245 261 10.1023/A:1026346121217

[B23] Nicot N Chiquet V Gandon B Amilhat L Legeai F Leroy P Bernard M Sourdille P Study of simple sequence repeat (SSR) markers from wheat expressed sequence tags (ESTs) Theoretical and Applied Genetics 2004 109 4 800 805 10.1007/s00122-004-1685-x 15146317

[B24] Jung S Abbott A Jesudurai C Tomkins J Main D Frequency, type, distribution and annotation of simple sequence repeats in Rosaceae ESTs Funct Integr Genomics 2005 5 3 136 143 10.1007/s10142-005-0139-0 15761705

[B25] Aggarwal RK Hendre PS Varshney RK Bhat PR Krishnakumar V Singh L Identification, characterization and utilization of EST-derived genic microsatellite markers for genome analyses of coffee and related species Theoretical and Applied Genetics 2007 114 2 359 372 10.1007/s00122-006-0440-x 17115127

[B26] Feng SP Li WG Huang HS Wang JY Wu YT Development, characterization and cross-species/genera transferability of EST-SSR markers for rubber tree (Hevea brasiliensis) Molecular Breeding 2009 23 1 85 97 10.1007/s11032-008-9216-0

[B27] Yonemaru J Ando T Mizubayashi T Kasuga S Matsumoto T Yano M Development of genome-wide simple sequence repeat markers using whole-genome shotgun sequences of sorghum (Sorghum bicolor (L.) Moench) DNA Res 2009 16 3 187 193 10.1093/dnares/dsp005 19363056 PMC2695772

[B28] Bedell JA Budiman MA Nunberg A Citek RW Robbins D Jones J Flick E Rholfing T Fries J Bradford K Sorghum genome sequencing by methylation filtration PLoS Biol 2005 3 1 e13 10.1371/journal.pbio.0030013 15660154 PMC539327

[B29] Yu JK Dake TM Singh S Benscher D Li W Gill B Sorrells ME Development and mapping of EST-derived simple sequence repeat markers for hexaploid wheat Genome 2004 47 5 805 818 10.1139/g04-057 15499395

[B30] Asp T Frei UK Didion T Nielsen KK Lubberstedt T Frequency, type, and distribution of EST-SSRs from three genotypes of Lolium perenne, and their conservation across orthologous sequences of Festuca arundinacea, Brachypodium distachyon, and Oryza sativa BMC Plant Biol 2007 7 36 10.1186/1471-2229-7-36 17626623 PMC1950305

[B31] Cardle L Ramsay L Milbourne D Macaulay M Marshall D Computational and experimental characterization of physically clustered simple sequence repeats in plants Genetics 2000 156 847 854 11014830 10.1093/genetics/156.2.847 PMC1461288

[B32] Lander ES Waterman MS Genomic mapping by fingerprinting random clones: a mathematical analysis Genomics 1988 2 3 231 239 10.1016/0888-7543(88)90007-9 3294162

[B33] Murray MG Palmer JD Cuellar RE Thompson WF Deoxyribonucleic acid sequence organisation in the mungbean genome Biochemistry 1979 18 5259 5266 10.1021/bi00590a034 497181

[B34] Seehalak W Somta P Sommanas W Srinives P Microsatellite markers for mungbean developed from sequence database Mol Ecol Resour 2009 9 862 864 10.1111/j.1755-0998.2009.02655.x 21564770

[B35] Sangsiri C Kaga A Tomooka N Vaughan D Srinives P Genetic diversity of the mungbean (*Vigna radiata*, Leguminosae) genepool based on microsatellite analysis Aust J Bot 2007 55 837 847 10.1071/BT07105

[B36] Provan J Powell W Hollingsworth PM Chloroplast microsatellites: new tools for studies in plant ecology and evolution Trends Ecol Evol 2001 16 142 147 10.1016/S0169-5347(00)02097-8 11179578

[B37] Wolfe KH Li WH Sharp PM Rates of nucleotide substitution vary greatly among plant mitochondrial, chloroplast, and nuclear DNAs Proc Natl Acad Sci USA 1987 84 24 9054 9058 10.1073/pnas.84.24.9054 3480529 PMC299690

[B38] Ouyang S Buell CR The TIGR plant repeat databases: a collective resource for the identification of repetitive sequences in plant Nucleic Acids Research 2004 32 360 363 10.1093/nar/gkh099 PMC308833 14681434

[B39] Somta P Seehalak W Srinives P Development, characterization and cross-species amplification of mungbean (*Vigna radiata*) genic microsatellite markers Conserv Genet 2009 http://www.springerlink.com/content/01738786115x452g/

[B40] Chaitieng B Kaga A Tomooka N Isemura T Kuroda Y Vaughan DA Development of a black gram [*Vigna mungo *(L.) Hepper] linkage map and its comparison with an azuki bean [*Vigna angularis *(Willd.) Ohwi and Ohashi] linkage map Theor Appl Genet 2006 113 7 1261 1269 10.1007/s00122-006-0380-5 16932883

[B41] Rohlf FJ NTSYS-pc: numerical taxonomy and multivariate analysis system version 2.2 2005 NewYork: Exeter Publishing Ltd16270455

[B42] Yano A Yasuda K Yamaguchi H A test for molecular identification of Japanese archaeological beans and phylogenetic relationship of wild and cultivated species of subgenus *Ceratotropis *(Genus *Vigna*, *Papilionaceae*) using sequence variation in two non-coding regions of the trnL and trnF genes Economic Botany 2004 58 S135 S146 10.1663/0013-0001(2004)58[S135:ATFMIO]2.0.CO;2

[B43] Ye TT Yamaguchi H Phylogenetic relationship of wild and cultivated *Vigna *(subgenus *Ceratotropis*, Fabaceae) from Myanmar based on sequence variations in non-coding regions of *trn*T-F Breed Sci 2007 57 271 280 10.1270/jsbbs.57.271

[B44] Tomooka N Yoon MS Doi K Kaga A Vaughan D AFLP analysis of diploid species in the genus Vigna subgenus Ceratotropis Genet Resour Crop Ev 2002 49 5 521 530 10.1023/A:1020954800107

[B45] Doi K Kaga A Tomooka N Vaughan DA Molecular phylogeny of genus Vigna subgenus Ceratotropis based on rDNA ITS and atpB-rbcL intergenic spacer of cpDNA sequences Genetica 2002 114 2 129 145 10.1023/A:1015158408227 12041826

[B46] Tomooka N Vaughan DA Moss H Maxted N The Asian *Vigna*: Genus *Vigna *Subgenus *Ceratotropis *Genetic Resources 2002 Kluwer, Dordrecht

[B47] Seehalak W Tomooka N Waranyuwat P Thipyapong P Laosuwan P Kaga A Vaughan DA Genetic diversity of the Vigna germplasm from Thailand and neighboring regions revealed by AFLP analysis Genet Resour Crop Evol 2006 53 1043 1059 10.1007/s10722-004-7939-2

[B48] Tomooka N Kaga A Vaughan D The Asian *Vigna *(*Vigna *subgenus *Ceratotropis*) biodiversity and evolution Plant Genome Diversity and Evolution 2006 Enfield: Science Publishers

[B49] Garza JC Slatkin M Freimer NB Microsatellite allele frequencies in humans and chimpanzees, with implications for constraints on allele size Mol Biol Evol 1995 12 4 594 603 7659015 10.1093/oxfordjournals.molbev.a040239

[B50] Lukashin AV Borodovsky M GeneMark.hmm: new solutions for gene finding Nucleic Acids Res 1998 26 4 1107 1115 10.1093/nar/26.4.1107 9461475 PMC147337

[B51] Conesa A Gotz S Garcia-Gomez JM Terol J Talon M Robles M Blast2GO: a universal tool for annotation, visualization and analysis in functional genomics research Bioinformatics 2005 21 18 3674 3676 10.1093/bioinformatics/bti610 16081474

[B52] Duvick J Fu A Muppirala U Sabharwal M Wilkerson MD Lawrence CJ Lushbough C Brendel V PlantGDB: a resource for comparative plant genomics Nucleic Acids Res 2008 36 Database D959 965 18063570 10.1093/nar/gkm1041 PMC2238959

[B53] Boutin SR Young ND Olson TC Yu ZH Vallejos CE Shoemaker RC Genome conservation among three legume genera detected with DNA markers Genome 1995 38 5 928 937 10.1139/g95-122 18470218

[B54] Choi HK Mun JH Kim DJ Zhu H Baek JM Mudge J Roe B Ellis N Doyle J Kiss GB Estimating genome conservation between crop and model legume species Proc Natl Acad Sci USA 2004 101 43 15289 15294 10.1073/pnas.0402251101 15489274 PMC524433

[B55] Huang TH Fan B Rothschild MF Hu ZL Li K Zhao SH MiRFinder: an improved approach and software implementation for genome-wide fast microRNA precursor scans Bmc Bioinformatics 2007 8 341 10.1186/1471-2105-8-341 17868480 PMC2206061

[B56] Ding Y Chan CY Lawrence CE Sfold web server for statistical folding and rational design of nucleic acids Nucleic Acids Res 2004 32 W135 W141 10.1093/nar/gkh449 15215366 PMC441587

[B57] Reinhart BJ Weinstein EG Rhoades MW Bartel B Bartel DP MicroRNAs in plants Genes Dev 2002 16 13 1616 1626 10.1101/gad.1004402 12101121 PMC186362

[B58] Sunkar R Zhu JK Novel and stress-regulated microRNAs and other small RNAs from Arabidopsis Plant Cell 2004 16 8 2001 2019 10.1105/tpc.104.022830 15258262 PMC519194

[B59] Molnar A Schwach F Studholme DJ Thuenemann EC Baulcombe DC miRNAs control gene expression in the single-cell alga *Chlamydomonas reinhardtii *Nature 2007 447 7148 1126 1129 10.1038/nature05903 17538623

[B60] Fattash I Voss B Reski R Hess WR Frank W Evidence for the rapid expansion of microRNA-mediated regulation in early land plant evolution BMC Plant Biol 2007 7 13 10.1186/1471-2229-7-13 17359535 PMC1838911

[B61] Murray MG Thompson WF Rapid isolation of high molecular weight plant DNA Nucleic Acids Res 1980 8 19 4321 4325 10.1093/nar/8.19.4321 7433111 PMC324241

[B62] Wang Z Weber JL Zhong G Tanksley SD Survey of plant short tandem repeats Theor Appl Genet 1994 88 1 6 10.1007/BF00222386 24185874

[B63] Lagercrantz U Ellegren H Andersson L The abundance of various polymorphic microsatellite motifs differs between plants and vertebrates Nucleic Acids Res 1993 21 5 1111 1115 10.1093/nar/21.5.1111 8464696 PMC309270

[B64] Rozen S Skaletsky H Primer3 on the WWW for general users and for biologist programmers Methods Mol Biol 2000 132 365 386 10547847 10.1385/1-59259-192-2:365

[B65] Botstein D White RL Skalnick MH Davies RW Construction of a genetic linkage map in man using restriction fragment length polymorphism Am J Hum Genet 1980 32 314 331 6247908 PMC1686077

[B66] Guo SW Thompson EA Performing the exact test of Hardy-Weinberg proportion for multiple alleles Biometrics 1992 48 2 361 372 10.2307/2532296 1637966

[B67] Liu K Muse SV PowerMarker: an integrated analysis environment for genetic marker analysis Bioinformatics 2005 21 9 2128 2129 10.1093/bioinformatics/bti282 15705655

