# Supplementary material for: Characterization of microsatellites and gene contents from genome shotgun sequences of mungbean (Vigna radiata (L.) Wilczek)
Source: BMC Plant Biol. 2009 Nov 24;9:137. doi: 10.1186/1471-2229-9-137 (PMC2788553; doi:10.1186/1471-2229-9-137)
Supplement: Additional file 1 — Characteristics of 58 primer pairs targeting 60 polymorphic microsatellite loci analyzed in 17 accessions of mungbean (16 cultivated and 1 wild mungbean) as listed in Additional File 3. PCR conditions and electrophoresis were described in Somta et al. (2008) [7]. Polymorphism information content (PIC), observed heterozygosity, expected heterozygosity, Hardy-Weinberg equilibrium (HWE), pair-wise and linkage disequilibrium (LD) of polymorphic loci were calculated using software POWERMARKER 3.25 [67]. [file 1471-2229-9-137-S1.DOC]

| **Table S1** Characteristics of 58 primer pairs targeting 60 polymorphic microsatellite loci analyzed in 17 accessions of mungbean. | | | | | | | | | | | | |
| --- | --- | --- | --- | --- | --- | --- | --- | --- | --- | --- | --- | --- |
| Marker name | Primer Name | Primer sequence | *N*aa | allele size range (bp) | *H*eb | *H*oc | *PIC*d | no. of reads per contig | motif | repeat no. | Matched accession no. | E-value |
| VR006 | VR006F | CCTTCTATCTCATGTTCACCGTC | 2 | 132-136 | 0.1172 | 0 | 0.1103 | 4 | ttta | 7 |  |  |
|  | VR006R | TGGAATAGGGACAAAATGGACT |  |  |  |  |  |  |  |  |  |  |
| VR029 | VR029F | GAAAGAAGCCAAACAAAACAGG | 2 | 158-161 | 0.1107 | 0 | 0.1046 | 8 | tag | 12 | emb|CAO64178.1|unnamed protein product [Vitis vinifera] | 4.00E-18 |
|  | VR029R | TGGCAGAGAAGGTAAATAAGGG |  |  |  |  |  |  |  |  |  |  |
| VR040 | VR040F | TGACAACATGGGAAGAAGAAGA | 4 | 167-182 | 0.7007 | 0.0588 | 0.6405 | 4 | gca | 11 |  |  |
|  | VR040R | ACACCAACACAAAAGCAAACAC |  |  |  |  |  |  |  |  |  |  |
| VR044 | VR044F | CCCATGAAGGTATGAGACAACA | 5 | 131-144 | 0.7356 | 0.1333 | 0.6907 | 3 | ag | 20 |  |  |
|  | VR044R | GACTGAGAAAGAGAGAGAAGCATTT |  |  |  |  |  |  |  |  |  |  |
| VR062 | VR062F | CGAAGACGAAATCTGAAGACAA | 3 | 130-148 | 0.5467 | 0 | 0.4486 | 4 | gaa | 7 |  |  |
|  | VR062R | TTACTTCTCCCAGCACTCCAAT |  |  |  |  |  |  |  |  |  |  |
| VR070 | VR070F | TGATTGTTGGAGAGTGCTCATT | 2 | 140-149 | 0.1107 | 0 | 0.1046 | 3 | tca | 9 |  |  |
|  | VR070R | CAATGTAGTTGATCCATCCGAA |  |  |  |  |  |  |  |  |  |  |
| VR073 | VR073F | GGTAGTTCATTTCGGCCACTT | 2 | 122-131 | 0.2076 | 0 | 0.1861 | 3 | cag | 10 | gb|ABK95988.1|unknown [Populus trichocarpa] | 4.00E-08 |
|  | VR073R | TATCTATGGTTTGCGAGCTGGT |  |  |  |  |  |  |  |  |  |  |
| VR078 | VR078F | CATGTGGCAACGCAGAAG | 2 | 133-136 | 0.1107 | 0 | 0.1046 | 2 | taa | 12 |  |  |
|  | VR078R | TCAACTTATTCCTCCTTTCTCTCAC |  |  |  |  |  |  |  |  |  |  |
| VR084 | VR084F | GAGCCACTTTGCCATATTTCT | 3 | 174-184 | 0.2145 | 0 | 0.2037 | 2 | ga | 17 |  |  |
|  | VR084R | ATTCTCCATTGTTCTCGTTCTC |  |  |  |  |  |  |  |  |  |  |
| VR086 | VR086F | GAGATCCTCCTACGGATTTGC | 2 | 106-108 | 0.1107 | 0 | 0.1046 | 5 | ga | 18 |  |  |
|  | VR086R | TTTCCTTCTCCAATTCTTGCTC |  |  |  |  |  |  |  |  |  |  |
| VR095 | VR095F | GAAATGGGAGTTCAAAGAGGAA | 2 | 115-121 | 0.375 | 0.125 | 0.3047 | 4 | tat | 8 |  |  |
|  | VR095R | TGGAGAAGTCTGGAAGAGAACC |  |  |  |  |  |  |  |  |  |  |
| VR099 | VR099F | ATACTTCGATCCGACCACTAGG | 4 | 190-287 | 0.519 | 0 | 0.4661 | 4 | ta | 14 |  |  |
|  | VR099R | CAAAGACAGGAGGAGAACAAGG |  |  |  |  |  |  |  |  |  |  |
| VR102 | VR0102F | CATGTGAGCTACCCTTTCAACA | 2 | 132-136 | 0.1107 | 0 | 0.1046 | 3 | ac | 10 | gb|AAY88919.1|pectinesterase [Olea europaea] | 2.00E-06 |
|  | VR0102R | CAAGGACTGCTATATCCAAGGC |  |  |  |  |  |  |  |  |  |  |
| VR108 | VR0108F | GCTCCAACACTCACTCACAAAC | 3 | 224-232 | 0.3443 | 0.0588 | 0.3125 | 2 | ttc | 9 |  |  |
|  | VR0108R | CAGAAATGCAGGAAAAGAGAGG |  |  |  |  |  |  |  |  |  |  |
| VR111 | VR0111F | TGCATCTTTATTGAGTTCCGTG | 4 | 158-182 | 0.6574 | 0 | 0.5976 | 10 | tctt | 7 |  |  |
|  | VR0111R | GTTTTGGGGTGAATGTTGGATA |  |  |  |  |  |  |  |  |  |  |
| VR133 | VR0133F | GAAGTGGCGGAAGATTGATAAG | 3 | 162-168 | 0.2145 | 0 | 0.2037 | 13 | tct | 7 |  |  |
|  | VR0133R | GGTAGATGGAAGGTAGAGGAATGA |  |  |  |  |  |  |  |  |  |  |
| VR135 | VR0135F | GCCCAGATTTGTTCATCCTAGA | 2 | 194-197 | 0.3599 | 0 | 0.2951 | 7 | tca | 7 |  |  |
|  | VR0135R | ACTGTTTTGAGTGGGGAAAAGA |  |  |  |  |  |  |  |  |  |  |
| VR140 | VR0140F | GGTGTTGTTGTTGAGGAATGAA | 2 | 184-186 | 0.4844 | 0 | 0.3671 | 5 | ta | 10 |  |  |
|  | VR0140R | AACATTGAGGACCCACATATCC |  |  |  |  |  |  |  |  |  |  |
| VR147 | VR0147F | CCATGTGTGTGAATGTGAGTGA | 2 | 102-104 | 0.1107 | 0 | 0.1046 | 2 | tg | 10 |  |  |
|  | VR0147R | CCTTTGATTTTGTGGGATGTGT |  |  |  |  |  |  |  |  |  |  |
| VR148 | VR0148F | CCGTTGTTGTTGCTGTTGTG | 2 | 148-158 | 0.1107 | 0 | 0.1046 | 2 | gtt | 18 |  |  |
|  | VR0148R | GAGCTTGCTAACCCTCTCCAAT |  |  |  |  |  |  |  |  |  |  |
| VR153 | VR0153F | AATTGTGAAGCAACAGAAAGCC | 3 | 146-154 | 0.3806 | 0 | 0.3399 | 4 | tgc | 7 |  |  |
|  | VR0153R | AGAAATAGGCAGGCAGTTTTCA |  |  |  |  |  |  |  |  |  |  |
| VR155 | VR0155F | AAGATCACACACAACCAACCC | 3 | 185-197 | 0.3045 | 0 | 0.2809 | 3 | ca | 10 |  |  |
|  | VR0155R | AATTAGTTCCACAGGCCAGATT |  |  |  |  |  |  |  |  |  |  |
| VR163 | VR0163F | AGGAGAAATTGTTGTTGTTCGG | 2 | 134-140 | 0.1107 | 0 | 0.1046 | 5 | caa | 8 |  |  |
|  | VR0163R | GTGTTGATTGTTAGGGAGGGAG |  |  |  |  |  |  |  |  |  |  |
| VR169 | VR0169F | GGAAGATAGCGGAGATGAAGAG | 3 | 132-138 | 0.5547 | 0 | 0.4555 | 5 | gt | 10 |  |  |
|  | VR0169R | CACCATACACCATAACATTCCTG |  |  |  |  |  |  |  |  |  |  |
| VR188 | VR0188F | ATACAAGGGCAGGTGTAGCATC | 2 | 224-226 | 0.1107 | 0 | 0.1046 | 11 | tc | 10 |  |  |
|  | VR0188R | CAGAAAACTTCATCCCCAGCTA |  |  |  |  |  |  |  |  |  |  |
| VR198 | VR0198F | AAGAAGAATGCGAGAAAGAAGC | 2 | 134-136 | 0.1107 | 0 | 0.1046 | 3 | ta | 10 |  |  |
|  | VR0198R | GTCCTAGAAGTTAGGGTTTGTGATT |  |  |  |  |  |  |  |  |  |  |
| VR200 | VR0200F | TGGGAAATAAAGAAAGCGTAGG | 2 | 106-114 | 0.1107 | 0 | 0.1046 | 2 | aga | 7 |  |  |
|  | VR0200R | CTCTTCTCCTTTGCCTCTACAAA |  |  |  |  |  |  |  |  |  |  |
| VR212 | VR0212F | AAACCAAAACGTAAGATCAGGG | 2 | 184-187 | 0.1107 | 0 | 0.1046 | 3 | taa | 7 |  |  |
|  | VR0212R | ATAGAAAGAAGTTGGCGCAGAA |  |  |  |  |  |  |  |  |  |  |
| VR216 | VR0216F | TTCCCTGTGTCCTTATATGTCC | 2 | 116-118 | 0.1107 | 0 | 0.1046 | 3 | ca | 10 | emb|CAO14811.1|unnamed protein product [Vitis vinifera] | 3.00E-15 |
|  | VR0216R | GAGGATAGTGAATTTTGAAGGC |  |  |  |  |  |  |  |  |  |  |
| VR222 | VR0222F | TCTCTTCTCTCTTCTCTCTTCTTCTTC | 5 | 142-170 | 0.3962 | 0.1176 | 0.3738 | 3 | tcta | 5 |  |  |
|  | VR0222R | TTGTGTCTGAGGCTATGTTGGT |  |  |  |  |  |  |  |  |  |  |
| VR223 | VR0223F | GCGTGATCGAGGCAGACTAT | 5 | 198-280 | 0.6074 | 0.0625 | 0.5574 | 2 | att | 7 |  |  |
|  | VR0223R | GTGGGTAGCTCGGTAATAGCAC |  |  |  |  |  |  |  |  |  |  |
| VR225 | VR0225F | CAGCAACAGAACTACAATCCCA | 3 | 150-156 | 0.5104 | 0.6471 | 0.4272 | 4 | ag | 12 |  |  |
|  | VR0225R | CGGCAATCCTCCTATATTCATT |  |  |  |  |  |  |  |  |  |  |
| VR226 | VR0226F | GCTTCTCTTTCTTGCATTCATC | 2 | 181-187 | 0.1107 | 0 | 0.1046 | 2 | ta | 11 |  |  |
|  | VR0226R | GACTAGGCGCTGGGAAAA |  |  |  |  |  |  |  |  |  |  |
| VR238 | VR0238F | ATTCTCTGCCTGCCATTTT | 2 | 137-146 | 0.1107 | 0 | 0.1046 | 2 | tc | 18 |  |  |
|  | VR0238R | ACGATTGTGTTTGTTGATGC |  |  |  |  |  |  |  |  |  |  |
| VR244 | VR0244F | GCTCTAAAACACGAAAGGGGT | 4 | 175-190 | 0.4141 | 0 | 0.3874 | 4 | ttc | 9 |  |  |
|  | VR0244R | TCATGGTGGAAGAAAAGCAA |  |  |  |  |  |  |  |  |  |  |
| VR248 | VR0248F | ATTCGGTTCCAGTGCTAAGAAG | 2 | 170-180 | 0.1107 | 0 | 0.1046 | 2 | ctt | 8 |  |  |
|  | VR0248R | AGCAGAAGTGCTTATCCCAGAG |  |  |  |  |  |  |  |  |  |  |
| VR256 | VR0256F | GCTGTGGTGTATTTACCTTGGG | 2 | 113-115 | 0.4688 | 0 | 0.3589 | 3 | tc | 11 | emb|CAO48648.1|unnamed protein product [Vitis vinifera] | 6.00E-11 |
|  | VR0256R | ATCCTCCGGTCATTATCTTGTG |  |  |  |  |  |  |  |  |  |  |
| VR257 | VR0257F | AGGAAGATGAGGGGAAAGTGA | 2 (locus 1) | 206-221 | 0.1107 | 0 | 0.1046 | 2 | gagt | 6 |  |  |
|  | VR0257R | TATTCTATACCTGCCACCCCAC | 6 (locus 2) | 152-176 | 0.609 | 0.1176 | 0.5769 |  |  |  |  |  |
| VR274 | VR0274F | ATTCGGGTAAAGTTCTGCATCT | 3 | 149-155 | 0.4031 | 0.0588 | 0.3422 | 2 | tat | 11 |  |  |
|  | VR0274R | AATGTTCACACACGTCATAGCA |  |  |  |  |  |  |  |  |  |  |
| VR293 | VR0293F | GTGGCTCACAAGGTAGTGCTAA | 2 | 222-225 | 0.2076 | 0 | 0.1861 | 4 | aag | 10 |  |  |
|  | VR0293R | GAGAGAAACAACCAACCAAAGG |  |  |  |  |  |  |  |  |  |  |
| VR303 | VR0303F | AGACGAAGAAGAAAACGCAGAC | 3 | 242-256 | 0.5952 | 0 | 0.5092 | 5 | gaaa | 5 |  |  |
|  | VR0303R | CCTCACACACAACACAACAGAA |  |  |  |  |  |  |  |  |  |  |
| VR304 | VR0304F | GAAGCGAAGAAGCCATAGAAAA | 4 | 170-188 | 0.7059 | 0 | 0.6516 | 5 | gaaa | 5 |  |  |
|  | VR0304R | CCTCACACACAACACAACAGAA |  |  |  |  |  |  |  |  |  |  |
| VR323 | VR0323F | ATATCAGCCATTGTTGCTTTCC | 2 | 176-180 | 0.1107 | 0 | 0.1046 | 4 | aatt | 5 | gb|ABK94936.1|unknown [Populus trichocarpa] | 1.00E-10 |
|  | VR0323R | TTCCCAGTTCAGACAACCAAGT |  |  |  |  |  |  |  |  |  |  |
| VR326 | VR0326F | GATGGCTCTGCATTGAAACC | 3 | 170-185 | 0.2145 | 0 | 0.2037 | 3 | agaga | 4 |  |  |
|  | VR0326R | GATCTTCCCAACTTTCCCTCTC |  |  |  |  |  |  |  |  |  |  |
| VR338 | VR0338F | ACTGAAGAGAATGGGTTAGGGG | 2 | 105-107 | 0.1107 | 0 | 0.1046 | 3 | ag | 14 |  |  |
|  | VR0338R | TCACATTTGTTGGGTTGAAGAG |  |  |  |  |  |  |  |  |  |  |
| VR357 | VR0357F | GCCCGATGTCCTAGCTTTTAG | 2 | 156-160 | 0.1107 | 0 | 0.1046 | 4 | attt | 5 |  |  |
|  | VR0357R | CCTCAAAACAATCAGAACTCTCG |  |  |  |  |  |  |  |  |  |  |
| VR361 | VR0361F | CTTGGACTTCGTCTCTGCG | 4 | 104-112 | 0.4141 | 0 | 0.3874 | 4 | aag | 7 |  |  |
|  | VR0361R | CAAAACAACCAACGCCATTAC |  |  |  |  |  |  |  |  |  |  |
| VR364 | VR0364F | GACGATTACTATGCCACCACAG | 2 | 194-200 | 0.1107 | 0 | 0.1046 | 2 | ttat | 5 |  |  |
|  | VR0364R | AATTCGAGAGAACCGACACAT |  |  |  |  |  |  |  |  |  |  |
| VR366 | VR0366F | GAATTTATTATCATCCGCACCC | 3 | 189-193 | 0.6172 | 0 | 0.5439 | 2 | ta | 16 |  |  |
|  | VR0366R | ATTGAAAAGACGGGAACAGCTA |  |  |  |  |  |  |  |  |  |  |
| VR375 | VR0375F | TCTCAGCATCTGTGGTGGTAGT | 3 | 166-173 | 0.1738 | 0.0625 | 0.1658 | 2 | gtt | 8 |  |  |
|  | VR0375R | AGAATCCAACAACTCCTGCTTC |  |  |  |  |  |  |  |  |  |  |
| VR390 | VR0390F | AGAATACAGAGAACCTGATACTTGGTC | 2 | 178-184 | 0.1107 | 0 | 0.1046 | 2 | gca | 7 | gb|ABW76288.1|beta-glucosidase G3 [Medicago truncatula] | 5.00E-19 |
|  | VR0390R | AATGGCTATGCAAATGGTAGGT |  |  |  |  |  |  |  |  |  |  |
| VR393 | VR0393F | TGGCACTTTCCATAACGAATAC | 2 | 148-152 | 0.4983 | 0 | 0.3741 | 3 | gat | 7 |  |  |
|  | VR0393R | ATCAGCCAAAAGCTCAGAAAAC |  |  |  |  |  |  |  |  |  |  |
| VR398 | VR0398F | TGCTAAAGGTTTCCTCTCAACT | 3 | 123-129 | 0.2612 | 0.1765 | 0.2448 | 2 | aga | 7 |  |  |
|  | VR0398R | GAATGAAGTCACGCACACAA |  |  |  |  |  |  |  |  |  |  |
| VR400 | VR0400F | ATCATAGATAGGGGACCAACCC | 2 (locus 1) | 149-152 | 0.0571 | 0.0588 | 0.0555 | 2 | cat | 8 |  |  |
|  | VR0400R | ATCTTAGGGAGTCTTCGAGGGA | 2 (locus 2) | 192-196 | 0.0571 | 0.0588 | 0.0555 |  |  |  |  |  |
| VR413 | VR0413F | GAGAAACCTTGGAGTTGGAGG | 3 | 98-104 | 0.3984 | 0 | 0.3542 | 3 | gtc | 8 |  |  |
|  | VR0413R | GCCTGTCAAGAAGGAACCTAAA |  |  |  |  |  |  |  |  |  |  |
| VR453 | VR0453F | TCTTTTCTATGTATGGCGCAAC | 2 | 255-257 | 0.1107 | 0 | 0.1046 | 3174 | ta | 13 | gb|ABV65140.1|ATP synthase CF1 beta subunit [Staphylea colchica] | 0 |
|  | VR0453R | TTGGCTTTCGTATTTCCTCAGT |  |  |  |  |  |  |  |  |  |  |
| VR468 | VR0468F | AGCTGCCCCTCTTTACTTAGATTT | 3 | 156-162 | 0.2145 | 0 | 0.2037 | 11 | ag | 10 |  |  |
|  | VR0468R | CGTCATTGCATACTTGAATTGG |  |  |  |  |  |  |  |  |  |  |
| VR487 | VR0487F | GGCAGGGAAGGAGGAAAA | 2 | 103-109 | 0.3599 | 0 | 0.2951 | 2 | tgt | 7 |  |  |
|  | VR0487R | CAGCCACAACAAGGCACA |  |  |  |  |  |  |  |  |  |  |
| Mean |  |  | 2.6833 |  | 0.2908 | 0.0289 | 0.2594 |  |  |  |  |  |
| a *N*a = Number of alleles, b *H*E = expected heterozygosity, c *H*O = Observed heterozygosity, d PIC = polymorphism information content | | | | | | | | | | |  |  |
