# Supplementary material for: Characterization of microsatellites and gene contents from genome shotgun sequences of mungbean (Vigna radiata (L.) Wilczek)
Source: BMC Plant Biol. 2009 Nov 24;9:137. doi: 10.1186/1471-2229-9-137 (PMC2788553; doi:10.1186/1471-2229-9-137)
Supplement: Additional file 2 — Dice's Similarity Matrix. This additional file contains a table expressing the Dice's Similarity Matrix. [file 1471-2229-9-137-S2.DOC]

| **Table S2: Dice's Similarity Matrix** | | | | | |  |  |  |  |  |  |  |  |  |  |  |  |  |  |  |  |  |  |  |  |  |  |  |  |  |  |  |  |  |  |
| --- | --- | --- | --- | --- | --- | --- | --- | --- | --- | --- | --- | --- | --- | --- | --- | --- | --- | --- | --- | --- | --- | --- | --- | --- | --- | --- | --- | --- | --- | --- | --- | --- | --- | --- | --- |
|  | **KPS1** | **KPS2** | **NM10-11-2** | **V2709** | **V2802** | **V3131** | **V6312** | **V1492** | **V4718** | **V1725** | **V1128** | **V2066** | **V6109** | **V6034** | **White Gold** | **Sukhothai** | **TPI25** | **ILRI114** | **JP109668** | **JP104332** | **AV71** | **JP72985** | **JP207984** | **JM2954** | **JP108515** | **JP107869** | **JP205884** | **JP108552** | **ILRI24953** | **JP107879** | **JP202462** | **JP100311** | **2004T2** | **JP107881** | **JP107876** |
| **KPS1** | 1.0000 |  |  |  |  |  |  |  |  |  |  |  |  |  |  |  |  |  |  |  |  |  |  |  |  |  |  |  |  |  |  |  |  |  |  |
| **KPS2** | 0.9091 | 1.0000 |  |  |  |  |  |  |  |  |  |  |  |  |  |  |  |  |  |  |  |  |  |  |  |  |  |  |  |  |  |  |  |  |  |
| **NM10-11-2** | 0.8261 | 0.8696 | 1.0000 |  |  |  |  |  |  |  |  |  |  |  |  |  |  |  |  |  |  |  |  |  |  |  |  |  |  |  |  |  |  |  |  |
| **V2709** | 0.7907 | 0.8837 | 0.8444 | 1.0000 |  |  |  |  |  |  |  |  |  |  |  |  |  |  |  |  |  |  |  |  |  |  |  |  |  |  |  |  |  |  |  |
| **V2802** | 0.8182 | 0.8636 | 0.9130 | 0.8372 | 1.0000 |  |  |  |  |  |  |  |  |  |  |  |  |  |  |  |  |  |  |  |  |  |  |  |  |  |  |  |  |  |  |
| **V3131** | 0.8837 | 0.8837 | 0.8000 | 0.8095 | 0.7907 | 1.0000 |  |  |  |  |  |  |  |  |  |  |  |  |  |  |  |  |  |  |  |  |  |  |  |  |  |  |  |  |  |
| **V6312** | 0.7907 | 0.7907 | 0.8889 | 0.9048 | 0.8837 | 0.8095 | 1.0000 |  |  |  |  |  |  |  |  |  |  |  |  |  |  |  |  |  |  |  |  |  |  |  |  |  |  |  |  |
| **V1492** | 0.8636 | 0.9091 | 0.8696 | 0.7907 | 0.9545 | 0.8372 | 0.8372 | 1.0000 |  |  |  |  |  |  |  |  |  |  |  |  |  |  |  |  |  |  |  |  |  |  |  |  |  |  |  |
| **V4718** | 0.8372 | 0.7442 | 0.7556 | 0.8571 | 0.7442 | 0.7619 | 0.8571 | 0.6977 | 1.0000 |  |  |  |  |  |  |  |  |  |  |  |  |  |  |  |  |  |  |  |  |  |  |  |  |  |  |
| **V1725** | 0.8182 | 0.8182 | 0.8261 | 0.9302 | 0.8182 | 0.8372 | 0.9302 | 0.7727 | 0.8837 | 1.0000 |  |  |  |  |  |  |  |  |  |  |  |  |  |  |  |  |  |  |  |  |  |  |  |  |  |
| **V1128** | 0.7111 | 0.8000 | 0.7234 | 0.7273 | 0.7111 | 0.7273 | 0.6364 | 0.7111 | 0.6818 | 0.6667 | 1.0000 |  |  |  |  |  |  |  |  |  |  |  |  |  |  |  |  |  |  |  |  |  |  |  |  |
| **V2066** | 0.8182 | 0.8636 | 0.8696 | 0.7907 | 0.9545 | 0.7907 | 0.8372 | 0.9545 | 0.6977 | 0.7727 | 0.7556 | 1.0000 |  |  |  |  |  |  |  |  |  |  |  |  |  |  |  |  |  |  |  |  |  |  |  |
| **V6109** | 0.8636 | 0.9091 | 0.7826 | 0.7907 | 0.8636 | 0.8372 | 0.7442 | 0.9091 | 0.6977 | 0.7727 | 0.7111 | 0.8636 | 1.0000 |  |  |  |  |  |  |  |  |  |  |  |  |  |  |  |  |  |  |  |  |  |  |
| **V6034** | 0.8000 | 0.8889 | 0.8085 | 0.8182 | 0.8000 | 0.7727 | 0.7273 | 0.8000 | 0.6818 | 0.7556 | 0.7391 | 0.8000 | 0.8444 | 1.0000 |  |  |  |  |  |  |  |  |  |  |  |  |  |  |  |  |  |  |  |  |  |
| **White Gold** | 0.8000 | 0.8444 | 0.8936 | 0.8182 | 0.9778 | 0.7727 | 0.8636 | 0.9333 | 0.7273 | 0.8000 | 0.6957 | 0.9333 | 0.8889 | 0.8261 | 1.0000 |  |  |  |  |  |  |  |  |  |  |  |  |  |  |  |  |  |  |  |  |
| **Sukhothai** | 0.7556 | 0.8444 | 0.8511 | 0.7727 | 0.8444 | 0.7273 | 0.7727 | 0.8444 | 0.6364 | 0.7111 | 0.7391 | 0.8889 | 0.8444 | 0.8261 | 0.8696 | 1.0000 |  |  |  |  |  |  |  |  |  |  |  |  |  |  |  |  |  |  |  |
| **TPI25** | 0.5714 | 0.5714 | 0.5000 | 0.5366 | 0.5238 | 0.5366 | 0.4878 | 0.5238 | 0.5366 | 0.5238 | 0.4651 | 0.5238 | 0.5714 | 0.5581 | 0.5116 | 0.5116 | 1.0000 |  |  |  |  |  |  |  |  |  |  |  |  |  |  |  |  |  |  |
| **ILRI114** | 0.2791 | 0.2791 | 0.3111 | 0.3333 | 0.3256 | 0.2857 | 0.3333 | 0.2791 | 0.3333 | 0.3721 | 0.2727 | 0.2791 | 0.2791 | 0.2273 | 0.3182 | 0.3182 | 0.1951 | 1.0000 |  |  |  |  |  |  |  |  |  |  |  |  |  |  |  |  |  |
| **JP109668** | 0.4186 | 0.4651 | 0.4444 | 0.5238 | 0.4651 | 0.4762 | 0.5238 | 0.4651 | 0.4762 | 0.5116 | 0.4545 | 0.5116 | 0.4651 | 0.4091 | 0.4545 | 0.5000 | 0.3415 | 0.2857 | 1.0000 |  |  |  |  |  |  |  |  |  |  |  |  |  |  |  |  |
| **JP104332** | 0.4000 | 0.3556 | 0.4255 | 0.4545 | 0.4000 | 0.3636 | 0.5000 | 0.3556 | 0.5000 | 0.5333 | 0.2609 | 0.3556 | 0.3556 | 0.3478 | 0.3913 | 0.3478 | 0.3256 | 0.2273 | 0.3636 | 1.0000 |  |  |  |  |  |  |  |  |  |  |  |  |  |  |  |
| **AV71** | 0.2727 | 0.2727 | 0.3043 | 0.3256 | 0.3182 | 0.2791 | 0.3721 | 0.3182 | 0.3256 | 0.3182 | 0.3111 | 0.3636 | 0.2727 | 0.2222 | 0.3111 | 0.3556 | 0.1905 | 0.2326 | 0.3721 | 0.3556 | 1.0000 |  |  |  |  |  |  |  |  |  |  |  |  |  |  |
| **JP72985** | 0.3721 | 0.3721 | 0.3556 | 0.2857 | 0.3256 | 0.3810 | 0.2857 | 0.3721 | 0.2381 | 0.2791 | 0.2727 | 0.3256 | 0.3721 | 0.2727 | 0.3182 | 0.3636 | 0.2439 | 0.3333 | 0.1905 | 0.1364 | 0.1860 | 1.0000 |  |  |  |  |  |  |  |  |  |  |  |  |  |
| **JP207984** | 0.3111 | 0.3111 | 0.3404 | 0.2727 | 0.3111 | 0.3636 | 0.2727 | 0.3111 | 0.3182 | 0.2667 | 0.4348 | 0.3556 | 0.3111 | 0.2609 | 0.3043 | 0.3478 | 0.3721 | 0.3636 | 0.4091 | 0.1739 | 0.2667 | 0.2727 | 1.0000 |  |  |  |  |  |  |  |  |  |  |  |  |
| **JM2954** | 0.2791 | 0.2791 | 0.2667 | 0.2381 | 0.2791 | 0.2857 | 0.2381 | 0.2791 | 0.1905 | 0.2326 | 0.2273 | 0.2791 | 0.2791 | 0.2727 | 0.2727 | 0.3182 | 0.2439 | 0.2857 | 0.1905 | 0.1364 | 0.2326 | 0.8571 | 0.1818 | 1.0000 |  |  |  |  |  |  |  |  |  |  |  |
| **JP108515** | 0.2791 | 0.2791 | 0.3111 | 0.2381 | 0.2791 | 0.2857 | 0.2381 | 0.2791 | 0.1905 | 0.2326 | 0.2727 | 0.3256 | 0.2791 | 0.2273 | 0.2727 | 0.3636 | 0.1951 | 0.3333 | 0.1905 | 0.0909 | 0.2326 | 0.7619 | 0.4091 | 0.6667 | 1.0000 |  |  |  |  |  |  |  |  |  |  |
| **JP107869** | 0.2667 | 0.2667 | 0.2979 | 0.3182 | 0.2667 | 0.2727 | 0.3182 | 0.2667 | 0.3182 | 0.3111 | 0.3043 | 0.3111 | 0.2667 | 0.2174 | 0.2609 | 0.3478 | 0.2326 | 0.3182 | 0.3636 | 0.1739 | 0.3111 | 0.7273 | 0.2609 | 0.6364 | 0.6818 | 1.0000 |  |  |  |  |  |  |  |  |  |
| **JP205884** | 0.2727 | 0.2727 | 0.2609 | 0.2326 | 0.2727 | 0.2791 | 0.2326 | 0.2727 | 0.1860 | 0.2273 | 0.3111 | 0.3182 | 0.2727 | 0.2222 | 0.2667 | 0.3111 | 0.1905 | 0.1860 | 0.2326 | 0.0889 | 0.2273 | 0.5116 | 0.2667 | 0.5581 | 0.6047 | 0.4889 | 1.0000 |  |  |  |  |  |  |  |  |
| **JP108552** | 0.2727 | 0.2727 | 0.2609 | 0.2326 | 0.2727 | 0.2791 | 0.2326 | 0.2727 | 0.1860 | 0.2273 | 0.2667 | 0.3182 | 0.2727 | 0.2222 | 0.2667 | 0.3556 | 0.2857 | 0.3256 | 0.2791 | 0.1778 | 0.2727 | 0.6977 | 0.4000 | 0.6512 | 0.6977 | 0.5778 | 0.5000 | 1.0000 |  |  |  |  |  |  |  |
| **ILRI24953** | 0.3478 | 0.3478 | 0.3333 | 0.3111 | 0.3478 | 0.3111 | 0.3111 | 0.3478 | 0.3111 | 0.3043 | 0.3830 | 0.3913 | 0.3478 | 0.2979 | 0.3404 | 0.4255 | 0.2727 | 0.3111 | 0.4000 | 0.1277 | 0.3478 | 0.1778 | 0.3404 | 0.2222 | 0.1778 | 0.2128 | 0.1739 | 0.3043 | 1.0000 |  |  |  |  |  |  |
| **JP107879** | 0.2791 | 0.2791 | 0.3111 | 0.3333 | 0.2791 | 0.2857 | 0.3333 | 0.2791 | 0.2857 | 0.3256 | 0.2273 | 0.2791 | 0.2791 | 0.2273 | 0.2727 | 0.3182 | 0.1951 | 0.2381 | 0.1905 | 0.1818 | 0.1860 | 0.6190 | 0.2273 | 0.5238 | 0.5714 | 0.5909 | 0.2791 | 0.4651 | 0.1778 | 1.0000 |  |  |  |  |  |
| **JP202462** | 0.2727 | 0.2727 | 0.2609 | 0.2791 | 0.2727 | 0.2791 | 0.2791 | 0.2727 | 0.2791 | 0.2727 | 0.2667 | 0.2727 | 0.2727 | 0.2222 | 0.2667 | 0.3111 | 0.1429 | 0.5581 | 0.3256 | 0.1333 | 0.2727 | 0.2326 | 0.3556 | 0.2791 | 0.2326 | 0.2222 | 0.1818 | 0.2273 | 0.4783 | 0.2326 | 1.0000 |  |  |  |  |
| **JP100311** | 0.3636 | 0.2727 | 0.3478 | 0.2791 | 0.3182 | 0.3256 | 0.3256 | 0.2727 | 0.3256 | 0.3182 | 0.2667 | 0.2727 | 0.2727 | 0.2222 | 0.3111 | 0.3111 | 0.1429 | 0.3721 | 0.1860 | 0.2222 | 0.2727 | 0.5581 | 0.2667 | 0.6047 | 0.6047 | 0.4444 | 0.5455 | 0.5455 | 0.2174 | 0.5116 | 0.3182 | 1.0000 |  |  |  |
| **2004T2** | 0.4000 | 0.3111 | 0.2979 | 0.2727 | 0.2667 | 0.3636 | 0.3182 | 0.3111 | 0.3182 | 0.3111 | 0.2174 | 0.2667 | 0.3111 | 0.2174 | 0.2609 | 0.3043 | 0.1395 | 0.2727 | 0.2273 | 0.1739 | 0.2222 | 0.5909 | 0.1739 | 0.5909 | 0.5000 | 0.5217 | 0.5333 | 0.4000 | 0.2128 | 0.4091 | 0.3111 | 0.6667 | 1.0000 |  |  |
| **JP107881** | 0.2791 | 0.2326 | 0.2222 | 0.1905 | 0.2326 | 0.2381 | 0.1905 | 0.2326 | 0.2381 | 0.1860 | 0.2273 | 0.2326 | 0.2326 | 0.1818 | 0.2273 | 0.2727 | 0.1463 | 0.2857 | 0.2381 | 0.1364 | 0.2326 | 0.6190 | 0.2273 | 0.6667 | 0.4762 | 0.5000 | 0.5116 | 0.4651 | 0.2222 | 0.3810 | 0.2791 | 0.6047 | 0.6818 | 1.0000 |  |
| **JP107876** | 0.4444 | 0.4889 | 0.4681 | 0.5455 | 0.4889 | 0.5000 | 0.5455 | 0.4889 | 0.5455 | 0.5333 | 0.4783 | 0.4889 | 0.4889 | 0.4348 | 0.4783 | 0.4783 | 0.3721 | 0.3182 | 0.6818 | 0.3043 | 0.3111 | 0.3636 | 0.4783 | 0.3182 | 0.2273 | 0.3913 | 0.2667 | 0.2667 | 0.3404 | 0.3182 | 0.3556 | 0.2222 | 0.3478 | 0.3636 | 1.0000 |
