# Supplementary material for: Characterization of microsatellites and gene contents from genome shotgun sequences of mungbean (Vigna radiata (L.) Wilczek)
Source: BMC Plant Biol. 2009 Nov 24;9:137. doi: 10.1186/1471-2229-9-137 (PMC2788553; doi:10.1186/1471-2229-9-137)
Supplement: Additional file 3 — Plants used in this study. This additional file contains a table listing all of the plants used in the study. [file 1471-2229-9-137-S3.DOC]

**Table S3**: Plants used in this study

| Accession/name | Code | Genus, subgenus, species | Race |
| --- | --- | --- | --- |
|  |  | Genus *Vigna*, subgenus *Ceratotropis* |  |
| JP104332 | Vac | *V. aconitifolia* | cultivated |
| JP72985 | Van | *V. angularis* var. *angularis* | cultivated |
| JM2954 | Van (wild) | *V. angularis* var. *nipponensis* | wild |
| AV71 | Var | *V. aridicola* | wild |
| JP205884 | Vex | *V. exilis* | wild |
| JP207984 | Vgr | *V. grandiflora* | wild |
| JP108515 | Vhi | *V. hirtella* | wild |
| JP107869 | Vmi | *V. minima* | wild |
| JP109668 | Vmu | *V. mungo* var. *mungo* | cultivated |
| JP107874 | Vmu (wild) | *V. mungo* var. *sylvestris* | wild |
| JP107879 | Vna | *V. nakashimae* | wild |
| JP107881 | Vne | *V. nepalensis* | wild |
| JP229096 | Vra | *V. radiata* var. *radiata* | cultivated |
| KPS1 | Vra | *V. radiata* var. *radiata* | cultivated |
| KPS2 | Vra | *V. radiata* var. *radiata* | cultivated |
| NM10-11-2 | Vra | *V. radiata* var. *radiata* | cultivated |
| V1128 | Vra | *V. radiata* var. *radiata* | cultivated |
| V1492 | Vra | *V. radiata* var. *radiata* | cultivated |
| V1725 | Vra | *V. radiata* var. *radiata* | cultivated |
| V2066 | Vra | *V. radiata* var. *radiata* | cultivated |
| V2709 | Vra | *V. radiata* var. *radiata* | cultivated |
| V2802 | Vra | *V. radiata* var. *radiata* | cultivated |
| V3131 | Vra | *V. radiata* var. *radiata* | cultivated |
| V4718 | Vra | *V. radiata* var. *radiata* | cultivated |
| V6034 | Vra | *V. radiata* var. *radiata* | cultivated |
| V6109 | Vra | *V. radiata* var. *radiata* | cultivated |
| V6312 | Vra | *V. radiata* var. *radiata* | cultivated |
| White gold | Vra | *V. radiata* var. *radiata* | cultivated |
| TPI25 | Vra (wild) | *V. radiata* var. *sublobata* | wild |
| ILRI 114 | Vst | *V. stipulacea* | wild |
| ILRI 24953 | Vsu | *V. subramaniana* | wild |
| JP108552 | Vte | *V. tenuicaulis* | wild |
| JP202462 | Vtr | *V. trilobata* | wild |
| JP100311 | Vum | *V. umbellata* | cultivated |
| 2004T2 | Vum (wild) | *V. umbellata* | wild |
|  |  | Genus *Vigna* subgenus *Vigna* |  |
| Songkla 1 | Vsn | *V. subterranea* | cultivated |
| JP202437 | Vun-Ung | *V. unguiculata* cv-gr. *Unguiculata* | wild |
| JP89083 | Vun-Ses | *V. unguiculata* cv-gr. *Sesquipedalis* | cultivated |
|  |  | Genus *Phaseolus* |  |
| PV042 | Pha | *P. vulgaris* | cultivated |
|  |  | Genus *Glycine* |  |
| AGS292 | Gly | *G. max* | cultivated |
