# Supplementary material for: Characterization of microsatellites and gene contents from genome shotgun sequences of mungbean (Vigna radiata (L.) Wilczek)
Source: BMC Plant Biol. 2009 Nov 24;9:137. doi: 10.1186/1471-2229-9-137 (PMC2788553; doi:10.1186/1471-2229-9-137)
Supplement: Additional file 4 — SSRs used in the phylogenetic study. This additional file contains a table showing the SSRs used in the phylogenetic study. [file 1471-2229-9-137-S4.DOC]

| **Table S4: SSRs used in phylogenetic study** | | | |
| --- | --- | --- | --- |
| **Number** | **SSR name** | **SSR primer** | **Primer sequence** |
| 1 | VR040 | VR040F | TGACAACATGGGAAGAAGAAGA |
|  |  | VR040F | ACACCAACACAAAAGCAAACAC |
| 2 | VR084 | VR084F | GAGCCACTTTGCCATATTTCT |
|  |  | VR084F | ATTCTCCATTGTTCTCGTTCTC |
| 3 | VR086 | VR086F | GAGATCCTCCTACGGATTTGC |
|  |  | VR086F | TTTCCTTCTCCAATTCTTGCTC |
| 4 | VR099 | VR099F | ATACTTCGATCCGACCACTAGG |
|  |  | VR099F | CAAAGACAGGAGGAGAACAAGG |
| 5 | VR102 | VR102F | CATGTGAGCTACCCTTTCAACA |
|  |  | VR102F | CAAGGACTGCTATATCCAAGGC |
| 6 | VR108 | VR108F | GCTCCAACACTCACTCACAAAC |
|  |  | VR108F | CAGAAATGCAGGAAAAGAGAGG |
| 7 | VR124 | VR124F | ACCCGAACCTGCATAAACATC |
|  |  | VR124F | ACCTATCTGAGTTGCATTTGGG |
| 8 | VR188 | VR188F | ATACAAGGGCAGGTGTAGCATC |
|  |  | VR188F | CAGAAAACTTCATCCCCAGCTA |
| 9 | VR222 | VR222F | TCTCTTCTCTCTTCTCTCTTCTTCTTC |
|  |  | VR222F | TTGTGTCTGAGGCTATGTTGGT |
| 10 | VR225 | VR225F | CAGCAACAGAACTACAATCCCA |
|  |  | VR225F | CGGCAATCCTCCTATATTCATT |
| 11 | VR274 | VR274F | ATTCGGGTAAAGTTCTGCATCT |
|  |  | VR274F | AATGTTCACACACGTCATAGCA |
| 12 | VR276 | VR276F | TTGATCCTTGTATTGGATGGTG |
|  |  | VR276F | GTGGGATTTCTGGTTTTGTTGT |
| 13 | VR304 | VR304F | GAAGCGAAGAAGCCATAGAAAA |
|  |  | VR304F | CCTCACACACAACACAACAGAA |
| 14 | VR357 | VR357F | GCCCGATGTCCTAGCTTTTAG |
|  |  | VR357F | CCTCAAAACAATCAGAACTCTCG |
| 15 | VR407 | VR407F | ACCCTGCAAAAGAAGTCGAA |
|  |  | VR407F | TACCATATCCACCACCGTTTCT |
| 16 | VR486 | VR486F | CGAGGGATCGGGTTAAGG |
|  |  | VR486F | ATGGATGAATTGAGTTGTTGGG |
